# A multicenter study on accuracy and reproducibility of nanopore sequencing-based genotyping of bacterial pathogens

**DOI:** 10.1128/jcm.00628-24

**Published:** 2024-08-19

**Authors:** Johanna Dabernig-Heinz, Mara Lohde, Martin Hölzer, Adriana Cabal, Rick Conzemius, Christian Brandt, Matthias Kohl, Sven Halbedel, Patrick Hyden, Martin A. Fischer, Ariane Pietzka, Beatriz Daza, Evgeny A. Idelevich, Anna Stöger, Karsten Becker, Stephan Fuchs, Werner Ruppitsch, Ivo Steinmetz, Christian Kohler, Gabriel E. Wagner

**Affiliations:** 1 Diagnostic and Research Institute of Hygiene, Microbiology and Environmental Medicine, Medical University of Graz, Graz, Austria; 2 Institute for Infectious Diseases and Infection Control, Jena University Hospital, Jena, Germany; 3 Genome Competence Center (MF1), Robert Koch Institute, Berlin, Germany; 4 Austrian Agency for Health and Food Safety, Vienna, Austria; 5 Ares Genetics GmbH, Vienna, Austria; 6 Medical and Life Sciences Faculty, Furtwangen University, Villingen-Schwenningen, Germany; 7 Nosocomial Pathogens and Antibiotic Resistances (FG13), Robert Koch Institute, Wernigerode, Germany; 8 Institute for Medical Microbiology and Hospital Hygiene, Otto von Guericke University Magdeburg, Magdeburg, Germany; 9 Enteropathogenic bacteria and Legionella (FG11), Consultant Laboratory for Listeria, Robert Koch Institute, Wernigerode, Germany; 10 Austrian Agency for Health and Food Safety, Graz, Austria; 11 Friedrich Loeffler Institute for Medical Microbiology, F.-Sauerbruch-Str., Greifswald, Germany; Cleveland Clinic, Cleveland, Ohio, USA

**Keywords:** nanopore sequencing, multicenter performance study, bacterial typing, genomic surveillance, cgMLST, molecular surveillance

## Abstract

Nanopore sequencing has shown the potential to democratize genomic pathogen surveillance due to its ease of use and low entry cost. However, recent genotyping studies showed discrepant results compared to gold-standard short-read sequencing. Furthermore, although essential for widespread application, the reproducibility of nanopore-only genotyping remains largely unresolved. In our multicenter performance study involving five laboratories, four public health-relevant bacterial species were sequenced with the latest R10.4.1 flow cells and V14 chemistry. Core genome MLST analysis of over 500 data sets revealed highly strain-specific typing errors in all species in each laboratory. Investigation of the methylation-related errors revealed consistent DNA motifs at error-prone sites across participants at read level. Depending on the frequency of incorrect target reads, this either leads to correct or incorrect typing, whereby only minimal frequency deviations can randomly determine the final result. PCR preamplification, recent basecalling model updates and an optimized polishing strategy notably diminished the non-reproducible typing. Our study highlights the potential for new errors to appear with each newly sequenced strain and lays the foundation for computational approaches to reduce such typing errors. In conclusion, our multicenter study shows the necessity for a new validation concept for nanopore sequencing-based, standardized bacterial typing, where single nucleotide accuracy is critical.

## INTRODUCTION

Genomic surveillance of microbial pathogens is crucial for modern infection control and hence for our health systems addressing the emergence (1), spread, and transmission of (new) pathogens (2, 3), drug-resistant strains (4), and vaccine-evading variants (5). As such, it plays a key role in data-driven decision-making and the implementation of countermeasures across clinical, animal health, and food safety sectors (4, 6–9). Ideally, surveillance involves global, long-term monitoring for population changes in circulating pathogens, coupled with local, (real-time) analysis to handle outbreaks swiftly, trace infection sources, and expedite patient management (6, 7). Whole-genome sequencing of pathogens by short-read (SR) next-generation sequencing has revolutionized the field (10, 11). This approach not only enabled investigations of complex relationships between different isolates at an unprecedented level (3, 6, 10), but it also tackled issues regarding standardization and reproducibility of existing methods (6). Additionally, the reconstructed genomes enable a reference-free, in-depth analysis of the genetic profiles of individual isolates, including factors associated with resistance, virulence, and pathogenicity (4, 6, 12).

Despite these advantages and their immense importance from a One Health viewpoint, widespread implementation and regular application of genomic surveillance via SR sequencing face hurdles such as high equipment cost, required laboratory space, low cost-efficiency with low sample numbers, complex bioinformatics workflows, and the need for trained personnel (8, 12).

The severe acute respiratory syndrome coronavirus 2 (SARS-CoV-2) pandemic, a prime example of the positive impact of extensive genomic surveillance and variant analysis (9), has shown how these limitations can be overcome through the use of straightforward protocols, the implementation of nanopore sequencing and automated, user-friendly analysis pipelines (13–16). Nanopore sequencing addressed the sequencing capacity bottleneck with its affordability, ease of implementation, and suitability for decentralized applications (3, 13, 14). Given the positive user experiences, the competitiveness with SR methods in SARS-CoV-2 surveillance (17) and the above-mentioned advantages, especially in combination with the real-time data availability (18) and selective sequencing (19, 20), its expansion into additional domains of routine surveillance is a logical next step for many.

This is enhanced by the fact that the increased error rates previously associated with nanopore sequencing compared to SR methods have been significantly reduced by recent advances in DNA library preparation chemistry (Q20+), flow cells (R10), and bioinformatics (basecalling models), resulting in a read accuracy above Q20 (21). In public health and clinical microbiology, routine nanopore sequencing holds promise in near-patient/real-time metagenomics for culture-free pathogen identification (22), plasmid characterization (20), and AMR marker screening (23). In the case of bacterial WGS, current studies show a very positive development of nanopore sequencing. Sereika et al. showed that recent nanopore advancements allow assemblies of a quality similar to those of Illumina data but with significantly less fragmentation. Lerminiaux et al. evaluated R10.4.1 flow cells and the V14 chemistry and showed that they yield high-quality and contiguous assemblies for Gram-negative bacteria, while finer-scale analyses on single nucleotide levels would still benefit from SR data (24). Another recent study with 14 different bacterial species has even shown that nanopore data using deep learning-based variant callers match or exceed Illumina data and are therefore excellently suited for variant calling approaches (25). However, during our study, there were also emerging reports of incorrect base calls attributed to methylation when sequencing native DNA from bacterial microorganisms (26, 27).

Classical genetic profiling/genetic typing seems particularly plausible as a near-term application of nanopore sequencing with considerable potential for the surveillance of bacterial pathogens (18). For in-depth genetic profiling and high-resolution typing of bacterial pathogens, e.g., for outbreak analysis, gene-by-gene approaches like core genome multilocus sequence typing (cgMLST) have become widely established offering high resolution in combination with standardization, ease of data exchange, and minimal hardware and bioinformatics knowledge requirements (28–31). Due to these properties, they have been extensively used in the field of genomic surveillance, especially in cross-laboratory/cross-national activities, e.g., the multicentric initiatives “PulseNet” and “Pathogenwatch.” In contrast to SNP analyses, alleles of a defined set of genes (e.g., the core genome) are called and the isolates are then characterized or compared on the basis of this genetic profile (28). Despite the continuously increasing raw read accuracy of nanopore sequencing and our promising results in the case of *Bordetella pertussis* typing (18), the reported results of other bacterial typing studies were discrepant (26, 32). Linde et al. reported good performance in plasmid assembly (32), an important advantage for analyzing AMR transmission (33, 34). They also report substantial problems for high-resolution genotyping in *Brucella suis* when comparing Illumina data to Oxford Nanopore Technologies (ONT) data from flow cell versions R9 and R10.4 (32). Similarly, Lohde et al. report unexpected sequencing errors of problematic magnitude that obscure typing results in *Klebsiella pneumoniae* and other species, which they attribute to nearby methylation sites causing systematic basecalling errors (26). Such errors can be highly problematic for allele-calling approaches, as by principle it is irrelevant whether one or one hundred bases change within a gene, both lead to the same result, namely to a different allele designation (28). Even a small number of erroneous SNPs when spread across random genes in the genome can accumulate incorrect allele calls and ultimately hinder analyses that demand high resolution, like outbreak analysis or source tracing investigations (26, 32).

These inconsistent results in the typing performance, particularly with regard to different species (18, 26, 32), underscore the necessity for systematic validation of nanopore sequencing in the domain of bacterial typing. Moreover, a crucial question, that largely lacks a definitive answer, pertains to the accuracy against gold-standard SR and especially the reproducibility of nanopore sequencing-based bacterial typing across diverse laboratories. This assessment is essential prior to implementing and establishing this innovative method in delicate domains like genomic surveillance and diagnostics.

A multicenter approach makes it possible to investigate whether the performance of the method and the reproducibility of the results are also guaranteed across various settings and thus enables a realistic assessment of the effectiveness and practical utility under real-world conditions.

In this study, we present the results of an international, multi-laboratory performance assessment aiming to thoroughly validate the use of nanopore sequencing for comprehensive genomic surveillance of bacterial pathogens in terms of typing accuracy and comparability. Using four healthcare-relevant species with 18–20 isolates each, we investigated the performance of nanopore R10.4.1 flow cells and V14 sequencing chemistry for routine high-resolution typing by cgMLST using data sets from five participants, including recognized public health institutions and established clinical microbiology laboratories.

## MATERIALS AND METHODS

### Strains and DNA isolation

Strains from four bacterial species comprising 19 *Enterococcus faecium* (EF), 20 *K. pneumoniae* (KP), 20 *Listeria monocytogenes* (LM), and 18 *Staphylococcus aureus* (SA) isolates were selected for this performance test. Of note, three additional isolates, EF21, SA64, and SA70, were only used as controls and were not analyzed in this study. Starting from a single colony and subsequent propagation, each laboratory (LAB1-5) was in a blind-coded manner provided with identical pure cultures of each strain in stitch-agar. Details on the cultivation of the strains and DNA preparation of the individual participants can be found in Table S1.

### Nanopore library preparation

The library preparation in all labs was carried out using the manufacturer’s ligation sequencing gDNA protocol using the Native Barcoding Kit 24 V14 SQK-NBD114.24 (Oxford Nanopore, UK). To investigate basecalling errors potentially resulting from DNA methylation 11 suspicious strains were resequenced in LAB1 according to the manufacturer’s ligation sequencing V14 — PCR barcoding protocol using the ligation kit SQK-LSK114 (Oxford Nanopore, UK) and its PCR-barcoding expansion EXP-PBC001 (Oxford Nanopore, UK).

### Nanopore sequencing

The initial sequencing procedure was performed on R10.4.1 flow cells run at 260 bp/s (4 kHz) using MinKNOW in all labs. Per run 10–20 strains were sequenced and the data were basecalled in super-accurate (SUP) mode. Lab-specific details including the number of multiplexed samples, DNA concentrations, and software versions used are denoted in Table S1. The PCR library (LAB1) was sequenced on R10.4.1 flow cells run at 400 bp/s (5 kHz) and basecalled in SUP mode using MinKNOW.

### Assembly pipeline

The nanopore reads were assembled with Flye (35) and assemblies were polished with Medaka (ONT) and its respective models: “r1041_e82_260bps_sup_g632” for the native barcoding kit data and “r1041_e82_400bps_sup_variant_v4.2.0” for the PCR barcoding data. Program versions are stated in Table S1.

### Performance study under improved sequencing conditions using a bacterial methylation basecalling model

For evaluation of recent sequencing advances made by the manufacturer during our study, LAB2 resequenced the same DNA preps again using the Native Barcoding kit as described above. Sequencing, however, was performed with the latest sequencing conditions using 400 bp/s and 5 kHz instead of the discontinued “accurate” mode and its 260 bp/s and 4 kHz. Raw data were basecalled with Dorado version 0.4.0 utilizing ONT’s specifically trained research model for bacterial methylation (res_dna_r10.4.1_e8.2_400bps_sup@2023-09-22_bacterial-methylation). Subsequently reads were assembled with Flye (35), and optional Racon (36) polishing was included to assess its impact on typing performance. Afterward, the Flye-only assembly as well as the Racon polished assembly were subjected to one round of Medaka polishing either with the recommended consensus model (-m r1041_e82_400bps_sup_v4.2.0) or with the variant model (-m r1041_e82_400bps_sup_variant_v4.2.0). cgMLST analysis was used to evaluate which of these four polishing variants delivered the best typing results. A subset of 12 strains (three for each species) was also resequenced in LAB1 and LAB3 to evaluate these updates in terms of reproducibility of typing results. Program versions are stated in Table S1.

### Short-read sequencing

Two independent short-read data sets were acquired for comparison with the long-read data sets. For the first short-read whole-genome sequencing data set, the same extracted DNA already used for long-read sequencing in Lab 2 was utilized for library preparation in the case of *S. aureus*, *K. pneumoniae,* and *E. faecium*. In the case of *L. monocytogenes*, an additional DNA preparation was used, but coming from a culture grown under the same culture conditions and from the same initial stock as described for the long-read DNA extraction. DNA libraries were generated using the Illumina DNA Prep Kit (Illumina, USA) combined with the Illumina DNA/RNA UD Indexes Set B and Tagmentation Kit (Illumina, USA). Five hundred nanograms of DNA were employed for the normalization process. Libraries were amplified using the Illumina DNA Prep PCR Kit (Illumina, USA) following the manufacturer’s protocol. All cleanup and size selection procedures were conducted using Illumina beads (Illumina, USA) according to the manufacturer’s guidelines. Library concentration was quantified using the Qubit 1X dsDNA Assay Kit (Thermo Fisher Scientific, USA). Sequencing was performed on the Illumina MiSeq DX with the MiSeq reagent Kit v3 2 × 300 Cycles (Illumina, USA) using 14 pM of prepared DNA library.

For the second independent short-read whole-genome sequencing data set, the starting material was the same culture as used for the DNA extraction for long-read sequencing in Lab 5. DNA was extracted with MagMAX Viral/Pathogen Ultra Nucleic Acid Isolation Kit (Thermofisher) on a KingFisher Apex robot. DNA libraries were generated with Illumina DNA prep Kit using the Illumina DNA/RNA UD Indexes (Sets A to D) and Tagmentation Kit (Illumina, USA). Between 100 to 500 ng of input DNA were used. Library concentration was quantified using the Qubit 1X dsDNA Assay Kit (Thermo Fisher Scientific, USA), and the fragment length of the library pool was measured in Qsep100 (Nippon Genetics). Sequencing was performed on an Illumina NextSeq 2000 device with the NextSeq 1000/2000 P1 Reagent cartridge of 300 Cycles (Illumina, USA) using 750 pM of prepared DNA library.

Subsequently, short-read genome assembly for Illumina data was carried out using the SKESA assembler version 2.4.0 (37), integrated into the Ridom SeqSphere+ version 9.0.8 (Ridom, Germany) (38).

### Typing with respective cgMLST schemes

All assemblies were analyzed with the cgMLST scheme for the respective species (28, 29, 39–41) in SeqSphere+. Typing results of the respective Illumina data served as a reference for allele calls of nanopore data. The exact number of mismatched cgMLST loci was calculated by cross-comparing all strains in distance matrices. Minimum spanning trees (MSTs) were built using default parameters to assess and visualize relations between assemblies of different laboratories, both between long-read (LR) data sets and between LR and SR. The parameter “pairwise ignore missing values” was selected in SeqSphere for all analyses.

### Exploration of the methylation error

A subset of suspicious strains with a high number of typing errors compared to the Illumina data were selected to thoroughly analyze reads and assemblies. Potential patterns of incorrect basecalls in reads covering erroneous loci were investigated via mapping of reads to reference alleles using minimap version 2.26 and visual inspection. To investigate patterns adjacent to ambiguous positions, and for the generation of the sequence logos the MPOA pipeline version 1.4.1 was applied as described previously (26). Seqtk version 1.3 (https://github.com/lh3/seqtk) with seed 99 and seed 100 was used for the downsampling of the data.

### Statistical testing

The assembly and polishing pipelines were compared by the Quade test followed by pairwise Wilcoxon signed rank tests as post hoc tests. The *P*-values of the post hoc tests were adjusted by Holm’s method. In all cases, results with an (adj.) *P*-values < 0.05 were considered significant.

## RESULTS

### Design and implementation of our nanopore validation study

We selected the species *E. faecium, K. pneumoniae, L. monocytogenes,* and *S. aureus* for our extensive validation due to their significant importance in the medical, public health, and food safety sectors. Moreover, whole-genome sequencing and genomic surveillance by cgMLST analysis are well-established and extensively used for these species (28, 29, 39–41). Each participating institution generated long-read sequencing data based on strictly defined conditions: Native barcoding kit V14 library prep, R10.4.1 flow cells, and basecalling in super accurate mode (SUP) for the highest data accuracy. Data sets from isolates with a sequencing depth below the recommended 40× (21) were excluded from further analysis (see Table S2). In accordance with ONT guidelines, assemblies were generated using Flye and polished with Medaka (42, 43). In total, we generated and analyzed 526 assemblies in our performance study (Table S2). Assembly metrics can be found in Table S3.

### cgMLST-based genotyping of short-read reference data sets shows consistent results

**Fig 1:**
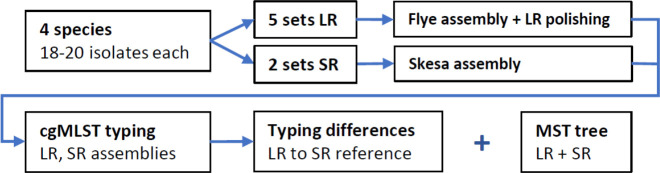
The methodological workflow of the study consists of LR and SR sequencing of 77 isolates. The assemblies of both methods were compared using cgMLST schemes for the respective species to assess differences in the typing results for the respective strain. In addition, the results were visualized in the form of MSTs containing assemblies of both methods.

The assemblies underwent high-resolution genotyping utilizing the respective cgMLST species schemes (28, 29, 39–41), followed by a comparative analysis. We specifically focused on a comparison of cgMLST results because they are pivotal in outbreak investigations, transmission analysis, and pinpointing infection sources. Two independent SR data sets facilitated a direct comparison with the established gold standard. The summarized methodological workflow is depicted in Fig. 1.

**Fig 2:**
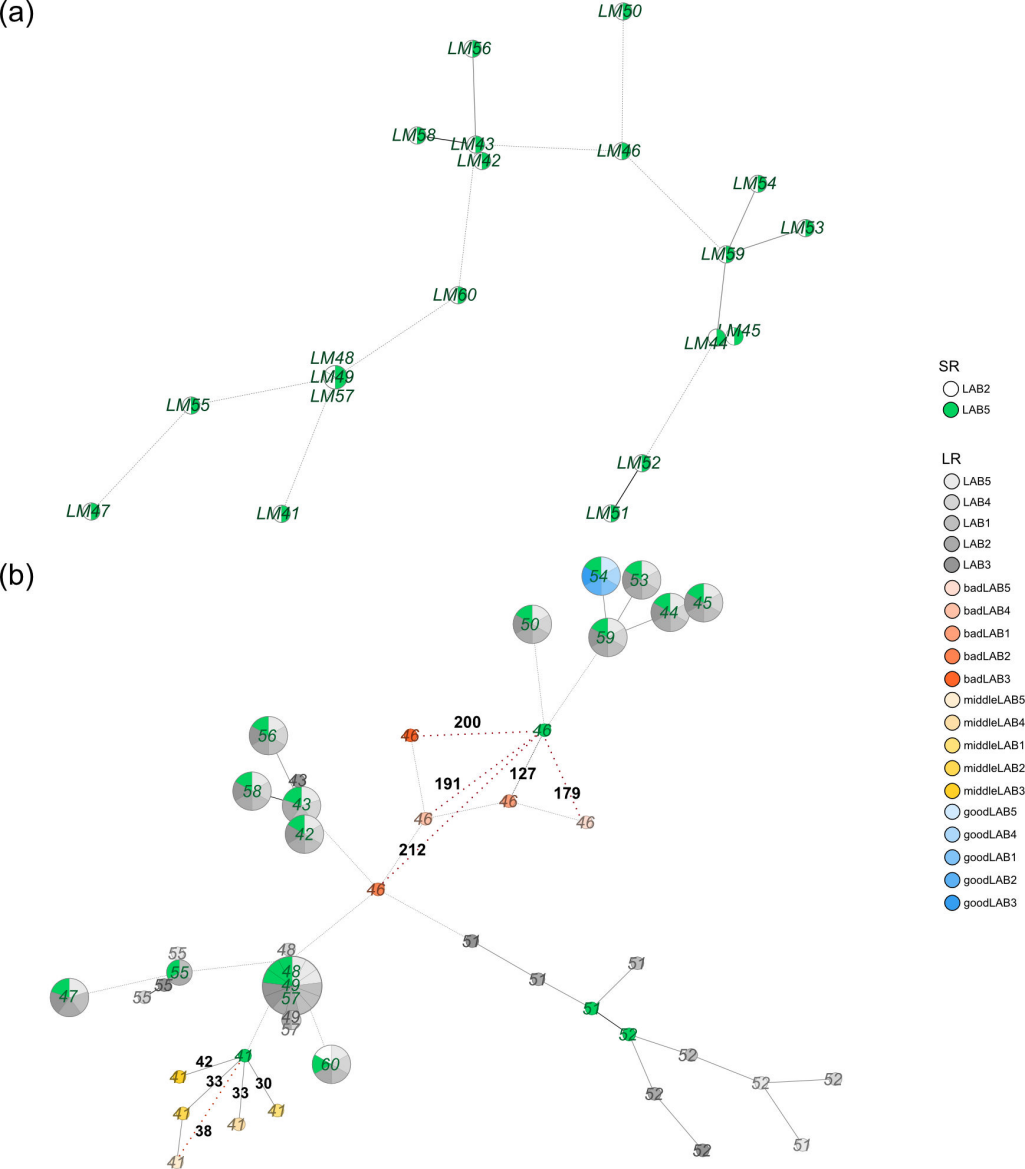
(**a**) cgMLST-based MST of *L. monocytogenes* isolates using short read data. Sequencing replicates of identical strains exhibit consistent cgMLST profiles, leading to direct clustering irrespectively of the executing laboratory. (**b**) MST of the same *L. monocytogenes* isolates, augmented with LR assemblies from participating laboratories (in grayscale). Only one SR data set is shown (in green). Depending on the strain under investigation, LR assemblies of different participants showed inconsistent typing results. There are isolates where the typing of the LR matches that of the SR (an exemplary one in blue shades), but also others with differences not only to the SR but also between the LR assemblies of the participants. Furthermore, the magnitude of the observed differences varied between isolates (an exemplary one in yellow and one in red shades). For a clearer presentation, we only show the differences for selected strains; the differences at the isolate level are detailed in Fig. 3.

In accordance with previous studies (44), the two SR data sets yielded consistent cgMLST results. In an MST, the sequencing replicates of distinct strains matched perfectly, as exemplified by *L. monocytogenes* (Fig. 2a). The same applies to the other species, as illustrated in Fig. S1a through c.

**Fig 3:**
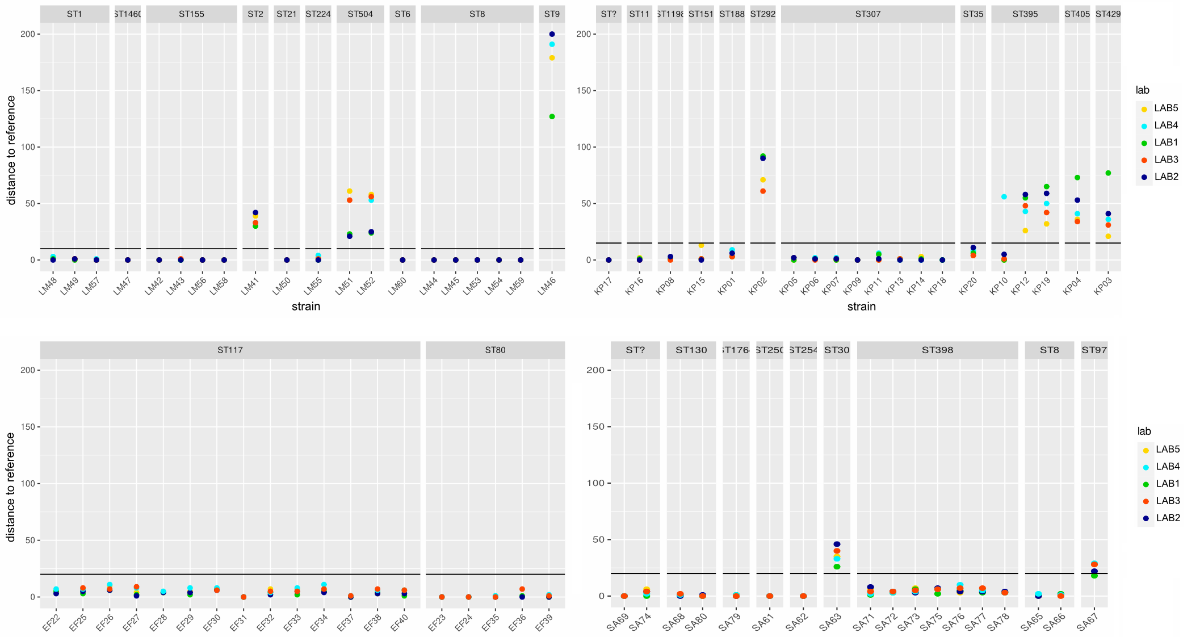
Allelic differences between the assemblies of the different participants compared to the short-read reference of the respective strains, showing species and isolate-specific differences regarding the number of affected strains and the magnitude of the differences. Isolates with the same ST show a similar error range compared to the assemblies of the individual participants. The cluster threshold for the respective cgMLST is added as a black line. Typing errors in assemblies that are close to this threshold are highly problematic in, e.g., outbreak investigations.

### Strain-specific inconsistencies revealed in cgMLST-based genotyping of long-read data sets, impeding typing correctness and reproducibility

The observations with LR data sets differed substantially from those with SR data. Although there were strains in which there were no/minimal differences in the typing results between the LR data of all participants and the SR reference, there were also strains where the typing of the LR data of different participants not only diverged from the reference but also from each other (Fig. 2b; Fig. S2 to S4). The magnitude of the observed typing errors varied depending on the strain (exemplary strains are shown in blue, yellow, and red shades in the figures). For each single isolate, the number of incorrect alleles compared to the reference was in a similar range for all participants, but the affected targets varied. This led to non-reproducible typing, clearly visible in the minimum spanning trees where it prevents the assemblies from coinciding in a single node. We could exclude the possibility that these observations arose from inappropriate extraction/handling of the DNA before its application to sequencing, as such issues would have similarly impacted the SR data and all isolates alike. Additionally, data from different labs still led to consistent LR typing results for several strains. Notably, LAB2’s SR and LR data sets originated from identical DNA preparation for *S. aureus*, *K. pneumoniae,* and *E. faecium*. Yet, discrepancies in typing were observed between the two datasets, comparable to those observed in other participants. This clearly shows that the typing errors are related to LR sequencing.

### Consistency in data quality from sequencing genetically similar strains

To assess the magnitude and variation of errors between participants for each isolate, we quantified the count of erroneous alleles across all assemblies in relation to their corresponding SR references. Based on the minimum spanning trees, we hypothesized that clusters of genetically similar isolates produced similar good/bad sequencing results and hence grouped the strains according to their MLST sequence type (ST). The analysis showed (Fig. 3) that there were differences between the species in (i) the number of strains affected and (ii) the extent of the observed error but also that (iii) the latter varied enormously between different isolates within a species.

Among all species, *L. monocytogenes*, e.g., had the highest number of isolates where the assemblies of all participants matched the reference perfectly but also included the strain with the highest number of differences to the short-read reference. It is also striking that strains with the same ST typically showed deviations from the reference of a similar magnitude between the participants. This indicates that the ST is indeed a good, first predictor of nanopore sequencing quality if strains with a certain ST have been previously sequenced and analyzed. In substantiating our hypothesis, we utilized our comprehensive collection of *Listeria* strains. We selected, sequenced, and typed three new isolates with previously good (Mismatches to the reference <3; ST1, ST8, ST155) and three isolates with previously poorly performing STs (Mismatches to the reference ≥3; ST2, ST9, ST504). As predicted, the former showed no (ST8, ST155) or minimal difference to the reference (ST1—1 difference), but the others showed 31 (ST2), 211 (ST9), and 62 (ST504) allelic differences.

Based on the criterion of ≥3 cgMLST mismatches (MM) compared to the SR reference in the majority of participants, we identified seven highly problematic isolates for *K. pneumoniae* (KP01, 02, 03, 04, 12, 19, 20), 12 for *E. faecium* (EF22, 25, 26, 27, 28, 29, 30, 32, 33, 34, 38, 40), four for *L. monocytogenes* (LM41, 46, 51, 52), and 10 for *S. aureus* (SA63, 67, 71, 72, 73, 74, 75, 76, 77, 78)—summarized in Table S4. For 10 additional isolates, at least one assembly of one participant was above the threshold, increasing the overall number of strains with at least one erroneous assembly to 43, which is more than half of the isolates. The criterion of ≥3 MM is based on the consideration that in investigations/studies where two problematic strains are involved, the genetic distance between them is artificially increased by at least six mismatches (at least three incorrect alleles in each isolate). Such an increase approaches the cluster threshold of cgMLST schemes in magnitude.

### Read-level analysis reveals base ambiguities due to wrong basecalls in the case of DNA methylation as a major source of error

To understand how the errors in the polished consensus assemblies arise, we examined the data at the read level by performing a mapping and subsequent visual inspection of the problematic sites in an initial screening of randomly selected targets in all species. At the position of the incorrect base that was responsible for the erroneous allele call, the mapping showed a base ambiguity that is not present in the short reads. One of the two ambiguous bases was predominantly found in forward mapping reads, while the other was primarily present in reverse mapping reads (Fig. S5a). This suggests errors associated with strand-specific methylation and is consistent with reports from other groups that emerged during our study (26, 27).

Interestingly, minimal differences in the data frequency of incorrect target reads between participants could influence the final typing result, even though the error position was conserved at the read level (Fig. S5). As an example given for the respective *E. faecium* reads of isolate EF26 at target “EF01658,” base frequencies—focusing only on the two prominent, ambiguous bases C and T that show a strand-bias—differed among participants: LAB1 (48% C—50%T), LAB2 (47% C—53%T), LAB3 (47% C—52%T), LAB4 (62% C—38%T), LAB5 (53% C—44%T). This influences which base appears at this position in an assembly’s sequence and thus finally determines the (non-reproducible) allele call.

**Fig 4:**
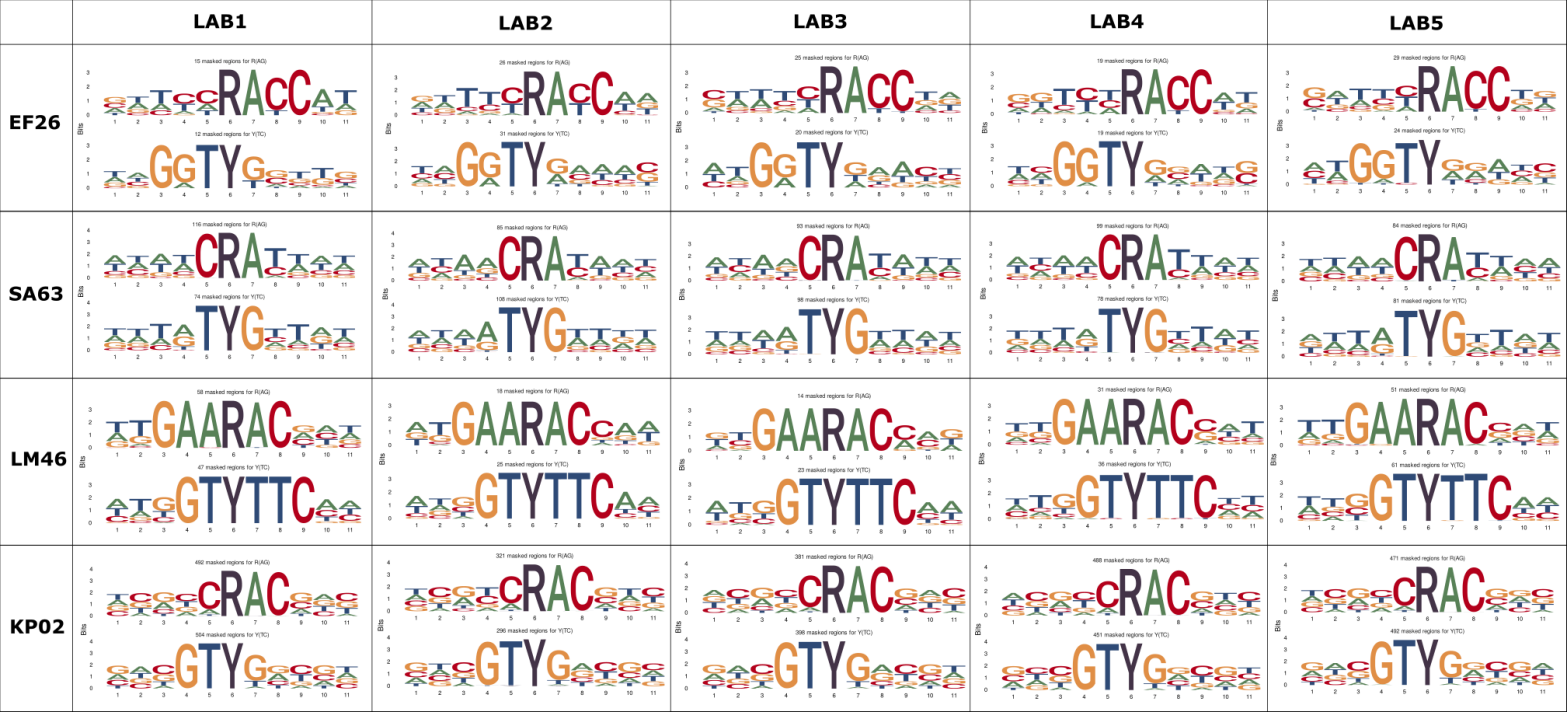
Sequence logos based on ambiguous positions and surrounding bases in a genome reveal conserved sequence patterns and high strain-level agreement between participants. Ambiguous sites identified were purine (R; A or G) or pyrimidine (Y; C or T) discrepancies.

Conserved sequence motifs also emerged when analyzing the surrounding positions around ambiguous bases using the MPOA pipeline (26). For this purpose, the reads of all participants of the most problematic strains, based on the cgMLST distance to the SR reference, were mapped to the respective assembly, ambiguous sites were identified and sequence logos were generated for the areas around ambiguous bases. As can be seen in Fig. 4, the patterns of the affected sites in the respective species were reproducible, again indicating a common cause of error.

The minimal differences in read counts between correct and incorrect bases suggest that random subsampling might slightly shift these ratios, affecting typing results. This was confirmed by subsampling into two datasets, showing that assemblies of well-performing strains remained consistent while problematic strains exhibited errors and typing discrepancies, highlighting data fragility (see Supplementary material for details).

Of note, we also investigated how the typing results are affected by the re-sequencing of the same DNA in the same lab. As expected due to the subsampling results, this did not have any significant influence on the typing results of good strains. In the case of problematic strains, however, also re-sequencing led to deviating and incorrect results (see Supplementary material and Tables S5 and S6 for details).

### PCR whole-genome amplification validates methylation as a predominant issue and resolves typing issues

As the observed errors strongly suggested issues arising from DNA methylation, we performed whole-genome amplification via PCR on some of the most problematic strains to remove DNA methylation. In line with our hypothesis, this approach significantly mitigated the observed issues in typing (Table 1). As previously observed (26, 27), however, in *K. pneumoniae*, it resulted in substantial assembly fragmentation (44–110 contigs), and consequently, a problematic increase in the number of missing targets in typing. Of 92 mismatching loci in the original KP02 assembly, 54 were correct in the PCR assembly and the remaining 38 were not found due to missing targets. Similarly, the mismatches of KP03 and KP12 were corrected in the PCR assemblies, with only one of those loci missing in the KP12 PCR assembly. In *E. faecium*, differences compared to the reference persisted in the low single digits, indicating additional sequencing errors not resolvable by PCR. However, as shown below, these errors could be corrected by updates in the basecalling model (Table S7), which is why we did not examine it in detail.

**TABLE 1:** Mismatches of problematic isolates compared to the short-read reference with and without PCR whole-genome-amplification reveals methylation as the most common source of sequencing errors affecting typing results^*a*^

	Native DNA Mismatches to reference	PCR amplified DNA Mismatches to reference
EF26	6	1
EF30	7	2
EF34	5	3
KP02	92	0 (994 missing)
KP03	77	1 (128 missing)
KP12	55	0 (89 missing)
LM41	30	0
LM46	127	0
LM51	23	0
SA67	18	0
SA73	6	0

^*a*^ Pronounced fragmentation of the PCR assembly and an elevated number of missing cgMLST targets was evident in *K. pneumoniae* (KP). Still, most of the mismatching targets from the original assembly (LAB1) were found and corrected in the respective PCR assembly (KP02 54/92, KP03 77/77, KP12 54/55—the other ones were missing in the PCR data sets).

### Sequencing updates improve typing performance, but challenges with problematic strains persist

**Fig 5:**
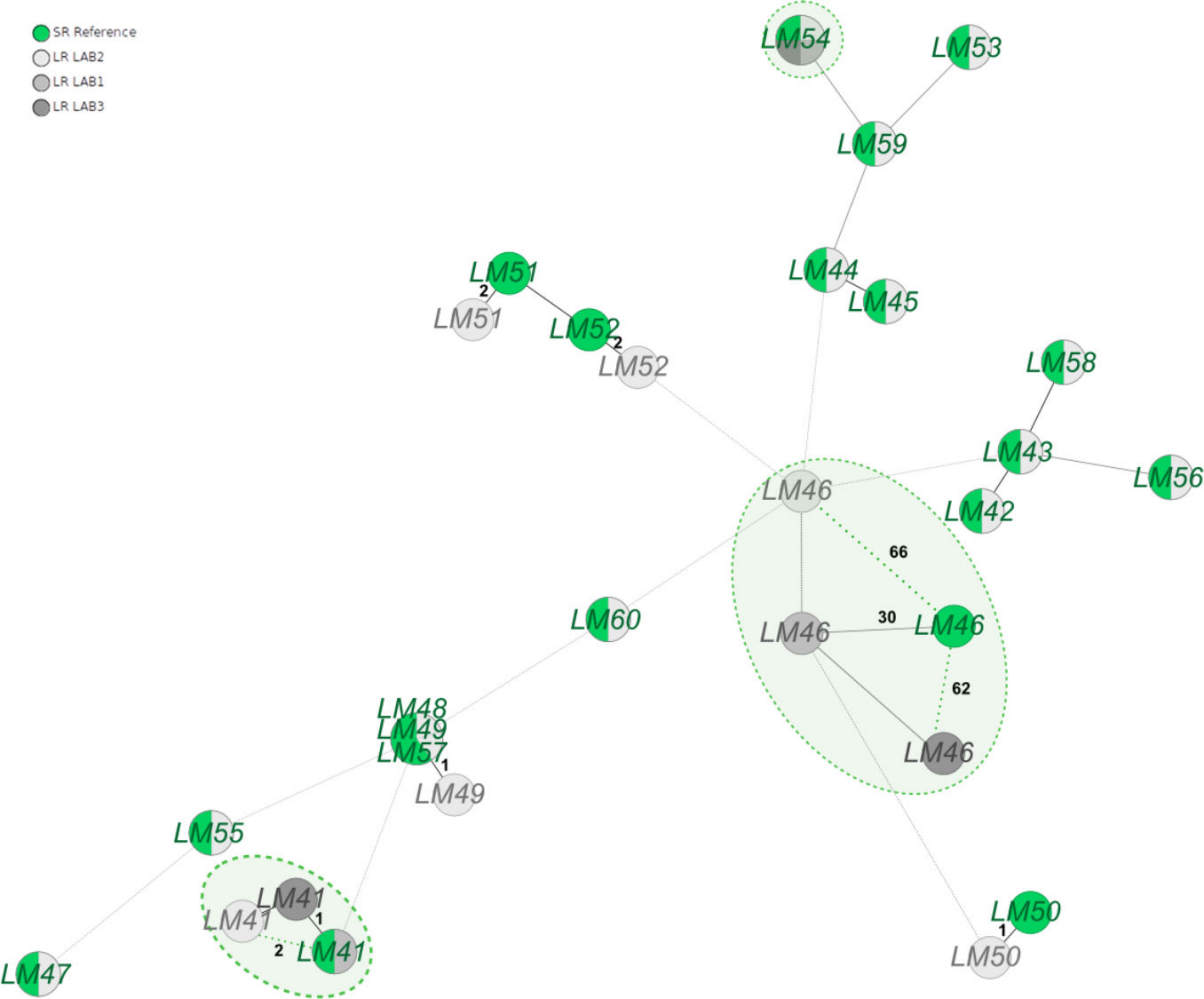
The MST of *L. monocytogenes* (LM), constructed using both SR (green) reference data and LR (in grayscale) data under the latest optimal conditions (sampling at 5 kHz, 400 bp/s, bacterial methylation model basecalling, polishing with Racon and Medaka using its variant model), vividly illustrates the significant enhancements achieved in nanopore sequencing. To further assess this progress, LR data from two additional participants were generated and analyzed for three selected isolates per species, encompassing one good and two challenging isolates (marked in circles). While this analysis highlights improvements, it also underscores the presence of errors that still pose challenges to the reproducibility of typing, as exemplified by strains like LM46. All distances from the re-sequenced LR assemblies to the reference SR assemblies not being zero are denoted in black numbers.

Throughout our investigation, the manufacturer implemented various enhancements, including a more precise data sampling process at an increased sequencing speed (5 kHz, 400 bp/s instead of 4 kHz, 260 bp/s) and the introduction of a dedicated research basecalling model tailored for native bacterial DNA and methylation (res_dna_r10.4.1_e8.2_400bps_sup@2023-09-22_bacterial-methylation). While a complete rerun of the performance test wasn't feasible, we aimed to evaluate these improvements by resequencing all isolates by one participant. Additionally, we explored different polishing strategies to identify the optimal tool combination for this updated sequencing data. Surprisingly, despite contrary official recommendations for bacterial assemblies (42, 43), the combination of the polishing tools Racon followed Medaka using its variant (!) polishing model (r1041_e82_400bps_sup_variant_v4.2.0) yielded the most accurate cgMLST typing results of the assemblies in our study. This tool combination performed significantly better according to the Quade test followed by pairwise Wilcoxon signed rank tests as post hoc test (Holm adj. *P*-value < 0.05) than pipelines with the non-variant Medaka mode or Flye-only assemblies without polishing (adj. *P*-values < 0.001) and had better but not significantly different results than Medaka variant without Racon (adj. *P*-value = 0.124) (Table S7). By applying the best pipeline only 4 of the 77 isolates have ≥3 mismatches compared to the SR reference, hence belonging to the problematic isolate category (Table S7). This is a decrease of 30 isolates compared to the initial results in the same laboratory. Notably, most LR assemblies now align seamlessly with the corresponding SR assemblies, as depicted in the MST in Fig. 5; Fig. S6 to S8.

Expanding upon our encouraging findings, we conducted a final multicenter assessment involving 12 selected isolates, comprising one good and two challenging isolates for each species. The enhancements were also clearly recognizable in this evaluation (refer to Fig. 5; Fig. S6 to S8; Table 2). However, it is noteworthy that the previously mentioned pattern of non-reproducibility of LR assemblies of problematic isolates, while significantly diminished, is still recognizable (LM46, KP04).

**TABLE 2:** Disparities in allelic variants within the LR assemblies of 12 selected and re-sequenced samples (sampling at 5 kHz, 400 bp/s, bacterial methylation model basecalling, polishing with Racon, and Medaka using its variant model) compared to the SR reference, with the improvements relative to the initial LR typing results noted in parentheses

	LAB1	LAB2	LAB3
EF22	1 (−4)	1 (−4)	2 (−8)
EF26	2 (−4)	0 (−4)	0 (−7)
EF35	1 (+1)	0 (−0)	0 (−0)
KP02	0 (−92)	1 (−89)	1 (−60)
KP04	37 (−36)	27 (−26)	17 (−17)
KP13	0 (−1)	0 (−0)	0 (−1)
LM41	0 (−30)	2 (−40)	1 (−32)
LM46	30 (−97)	66 (−134)	62 (−150)
LM54	0 (−0)	0 (−0)	0 (−0)
SA62	0 (−0)	0 (−0)	0 (−0)
SA63	2 (−24)	1 (−45)	1 (−39)
SA67	0 (−18)	1 (−21)	0 (−28)

## DISCUSSION

Nanopore-based whole-genome sequencing of bacteria increasingly finds its way into genomic surveillance of pathogens and has the potential to revolutionize the field due to its low cost and simple application. However, our study demonstrated that, in the case of high-resolution typing of bacteria nanopore sequencing does not yet achieve uniform sequencing quality across strains and reproducibility between labs compared to gold-standard SR methods. For the latter, in agreement with previous studies (44), all replicates of individual strains in different laboratories provided virtually identical results in the case of standardized and high-resolution cgMLST analysis (compare Fig. 2 or Fig. S1 to S4). Remarkably, the discrepancies between long-read typing results from different participants of the same isolate were in some cases near or even surpassing the cgMLST scheme’s cluster threshold for assigning isolates to outbreak clusters (see Fig. 3). Since it is not guaranteed that related isolates or even replicates of an individual isolate can be assigned to the same outbreak cluster, the method presently might not meet the requirements for high-resolution typing in such a critical setting. Furthermore, the issue of reproducibility in typing results for problematic isolates would also have an impact on cross-laboratory and cross-national surveillance.

The problem was significant, as all four studied species with public health relevance were affected to a certain degree - with the number of affected isolates varying by the species. Even within a single species, the frequency of errors varied between isolates, as the range spanned from none to several hundred incorrect alleles compared to the reference, indicating the origin of the problem at the strain level. The issue did not occur randomly. Our multicenter analyses showed that genetically similar strains, sharing the sequence type (ST), typically provided similar typing results and error ranges (Fig. 3). The ST might therefore be used for an initial estimate of data quality if comparative data is available. Particular caution should be taken when interpreting data from new or problematic STs.

Our results also clarify the discrepant results in the literature concerning the accuracy of the recent Nanopore Q20+ chemistry, when applied to a given bacterial species (28, 29, 39–41). It is important to realize that not only the species but also the strain(s) under investigation are relevant. Although results may appear contradictory at first, they might result from this (technical) limitation. Contrary to SR technologies, high typing quality of one strain (and species) did not guarantee success with other isolates from the same species in LR sequencing (see Fig. 2b and 3; Fig. S2 to S4). Consequently, general conclusions should be drawn cautiously, especially when studies and observations are based on only one or a few strains per species. In contrast to SR sequencing, nanopore sequencing would require a fundamentally different approach to performance studies of high-resolution genotyping: the crucial factor lies not solely in a high number of strains but primarily in achieving a high genetic diversity in strains and species to ensure sampling of problematic strains. This is evident in our *L. monocytogenes* strain collection. The 15 strains, in which the typing of all participants showed negligible differences to the reference, were contrasted with the most problematic isolate of the entire performance study, LM46. Conversely, our results of *E. faecium* isolates were generally good, but diversity was low (only covering two STs). It is conceivable that more severely affected strains may also be present within this species.

Upon analyzing the reads of problematic strains, consistency emerged, revealing similar error-prone sequence patterns (Fig. 4) and strand-specific basecalling errors (Fig. S5) in the sequencing data of the participants. The plausible explanation that these problems arise from DNA methylation, as reported in other recent studies (26, 27), could be confirmed by whole-genome PCR amplification, which largely resolved the typing problems (see Table 1). Apart from the fact, that this approach requires additional wet lab work, PCR also introduces new challenges (26, 27, 45), such as increased cost, issues in amplifying GC-rich species, the introduction of new errors due to amplification and shorter reads resulting in less contiguous *de novo* assemblies and, subsequently, an increased number of missing targets in cgMLST analysis (Table 1).

A problem with far-reaching implications only became apparent through this study’s multicenter approach. Although the identified methylation issue in cgMLST typing resulted in a similar order of magnitude of incorrect alleles in individual isolates between participants (Fig. 3), the affected targets varied. The difference between the correctly or the incorrectly called alleles of a target was typically reflected in a single different (incorrect) base. Even minimal differences in the data sets (Fig. S5), either a few additional reads with the correct or the incorrect base—could ultimately have significant implications on the allelic typing being correct or incorrect in the final assemblies. If the error related to methylation accumulates over several targets, it leads to completely non-reproducible allele profiles of individual isolates, which has a detrimental effect on the downstream analysis (Fig. 2b; Fig. S2 to S4). To exclude a contribution from other sources of error, we randomly subsampled data sets, and even these subsamples resulted in different allelic profiles compared to each other (Table S5), illustrating the inherent fragility of the read data. Consequently, our investigation highlights the potential for detecting problematic isolates by sequencing biological replicates or merely subsampling the data, provided that the sequencing depth permits.

The consistent errors in reads found among participants suggest the possibility and need for technical and software interventions to address the issue. Indeed, technical improvements during our study and our optimization of the polishing strategy improved typing, nevertheless, there were still problematic results for four isolates (KP03, KP04, KP12, and LM46). While this number may seem low, it is reasonable to assume additional cases, as the strains examined in our study represent only a minimal fraction of the population of a given species. This is complicated by the fact that in, e.g., outbreak scenarios, one is dealing with genetically similar strains. Should the error manifest in one strain, it is likely to recur in others.

Thus, the application of nanopore sequencing in high-resolution typing, e.g., for outbreak analysis or source tracings, must be evaluated critically currently. The potential for inaccurate data and flawed genotyping, which could have significant implications for decision-making, is deemed too high, even when dealing with a limited number of affected strains.

Nevertheless, the potential of nanopore sequencing is evident in our study. When the DNA modifications did not interfere, sequencing and typing of unproblematic strains yielded consistently good results across all participants. These results were robust despite minimal differences in laboratory workflows, a characteristic crucial for widespread applicability.

Although our study still shows a clear need for action, the substantial enhancements made by the manufacturer recently instill optimism that further developments can resolve the issue. The main challenge will be to ensure robust performance even for emerging strains with novel patterns of DNA modifications that are not represented in the models. Furthermore, it would be beneficial to conduct a detailed investigation of the specific characteristics that differentiate the “problematic” strains from the others in a future study. This should entail examining whether there are differences in the frequency of methylation and whether genetic markers can be used to identify such strains.

Since major improvements are based on models for basecalling and polishing, trained using machine learning, open communication of the training data set is required for a systematic evaluation to exclude a training bias, as observed for many A.I. models. Ideally, the improvements should apply to the entire species population and not just to a subgroup (newly integrated into the training set). Furthermore, to ensure improved but consistent results with previous iterations and future applications in routine genomic surveillance, quality control parameters need to be developed and published together with details about the new models. Besides the species and strain composition used for the model training, this should also include how and to what extent the models were validated.

### Conclusion

As impressive as nanopore sequencing performs in many areas, it is not yet generally applicable in the clinical/public health sector for high-resolution bacterial genotyping, where even a few incorrect bases infer with the analysis, especially given the massive consequences this can ultimately have. Because the observed problem of incorrect basecalls is highly isolate-dependent even within a given species, we argue that future improvements must be evaluated using strain collections of a given species with an emphasis on a high genetic diversity. Based on our observations, we also recommend including parameters such as read ambiguity at specific positions, agreement between forward and reverse reads, and analysis of sequencing replicates for comprehensive quality assessment of nanopore data. Furthermore, for the basecalling and polishing models, the strains of the training data set as well as their quality control parameters should be disclosed to ensure a systematic evaluation and subsequently a safe use in genomic pathogen monitoring.

## Data Availability

Nanopore and Illumina read data sets of all participants have been deposited under BioProject accession no. PRJNA1091452 in the National Center for Biotechnology Information Sequence Read Archive repository.

## SUPPLEMENTAL MATERIAL

Supplemental material jcm.00628-24-s0001.pdf Figures S1 to S8; Tables S5 and S6.

Table S1 jcm.00628-24-s0002.xlsx Experimental details.

Table S2 jcm.00628-24-s0003.xlsx Information about obtained sequencing data.

Table S3 jcm.00628-24-s0004.xlsx Assembly metrics.

Table S4 jcm.00628-24-s0005.xlsx Typing differences for problematic strains.

Table S7 jcm.00628-24-s0006.xlsx Distance to the reference for best pipeline for updated assemblies.

ASM does not own the copyrights to Supplemental Material that may be linked to, or accessed through, an article. The authors have granted ASM a non-exclusive, world-wide license to publish the Supplemental Material files. Please contact the corresponding author directly for reuse.
