# Supplementary material for: A multicenter study on accuracy and reproducibility of nanopore sequencing-based genotyping of bacterial pathogens
Source: J Clin Microbiol. 2024 Aug 19;62(9):e00628-24. doi: 10.1128/jcm.00628-24 (PMC11389150; doi:10.1128/jcm.00628-24)
Supplement: Supplemental material — Figures S1 to S8; Tables S5 and S6. [file jcm.00628-24-s0001.pdf]

A multicenter study on accuracy and reproducibility of nanopore sequencing-based genotyping of bacterial pathogens

Johanna Dabernig-Heinz[1*], Mara Lohde[2*], Martin Hölzer[3*], Adriana Cabal[4*], Rick Conzemius[5*], Christian Brandt[2*], Matthias Kohl[6], Sven Halbedel[7,8], Patrick Hyden[4], Martin A. Fischer[9], Ariane Pietzka[10], Beatriz Daza[4], Evgeny A. Idelevich[11], Anna Stöger[4], Karsten Becker[11], Stephan Fuchs[3*], Werner Ruppitsch[4*], Ivo Steinmetz[1#], Christian Kohler[11#,*], & Gabriel E. Wagner[1#,*]

* These authors contributed equally # Shared corresponding authors

[1] Diagnostic and Research Institute of Hygiene, Microbiology and Environmental Medicine, Medical University of Graz, Neue Stiftingtalstraße 6, 8010 Graz, Austria.
[2] Institute for Infectious Diseases and Infection Control, Jena University Hospital, Am Klinikum 1, 07747 Jena, Germany.
[3] Genome Competence Center (MF1), Robert Koch Institute, Seestraße 10, 13353 Berlin, Germany.
[4] Austrian Agency for Health and Food Safety, Währingerstraße 25a, 1096 Vienna, Austria
[5] Ares Genetics GmbH, Carlbergergasse 66, 1230 Vienna, Austria.
[6] Medical and Life Sciences Faculty, Furtwangen University, 78054 Villingen-Schwenningen, Germany.
[7] Nosocomial Pathogens and Antibiotic Resistances (FG13), Robert Koch Institute, Burgstrasse 37, 38855 Wernigerode, Germany
[8] Institute for Medical Microbiology and Hospital Hygiene, Otto von Guericke University Magdeburg, Leipziger Str. 44, 39120 Magdeburg, Germany.
[9] Enteropathogenic bacteria and Legionella (FG11), Consultant Laboratory for Listeria, Robert Koch Institute, Burgstrasse 37, 38855 Wernigerode, Germany
[10] Austrian Agency for Health and Food Safety, Beethovenstraße 6, 8010 Graz, Austria
[11] Friedrich Loeffler Institute for Medical Microbiology, F.-Sauerbruch-Str., 17475 Greifswald, Germany.

Results

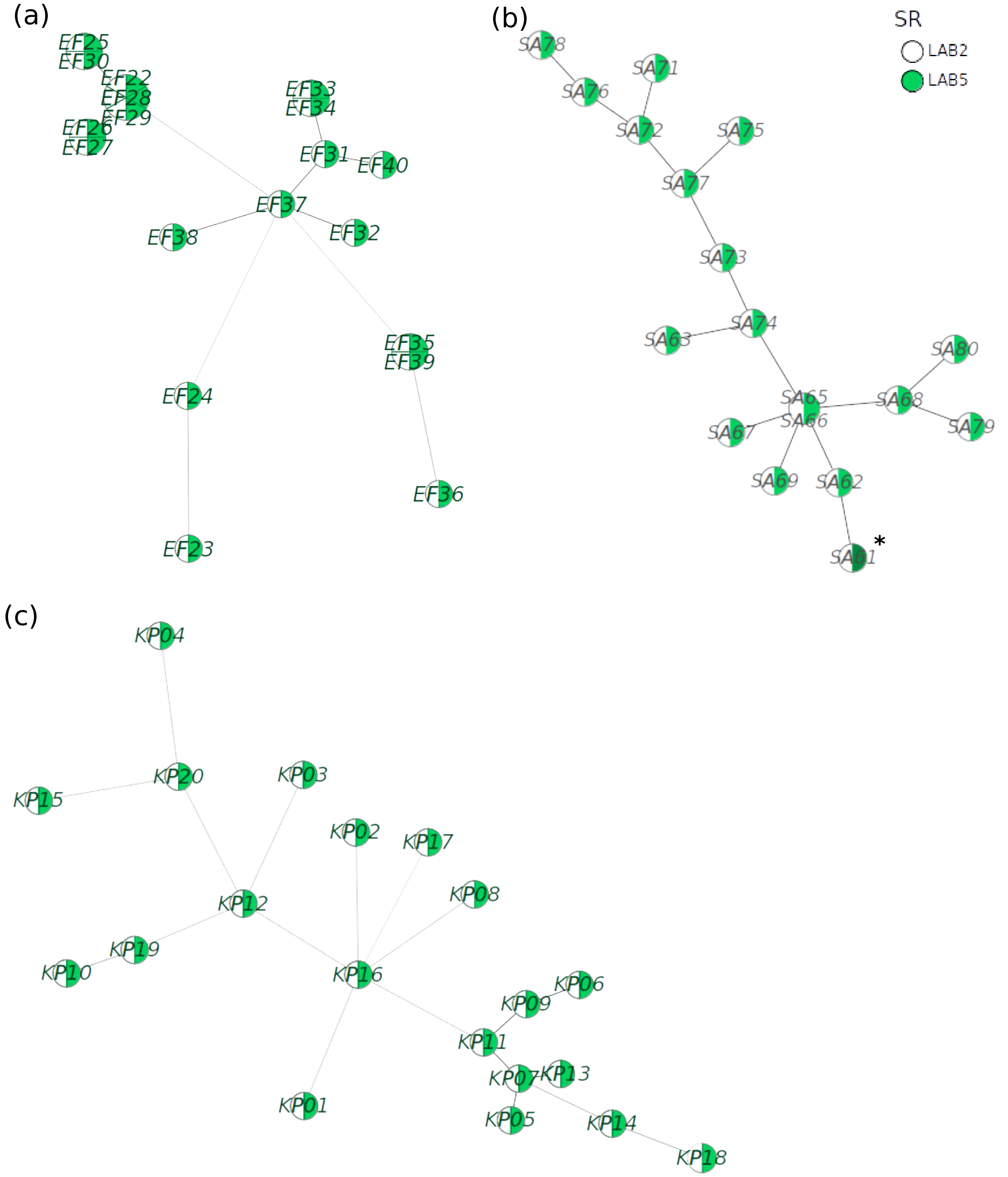

Supplementary Figure 1: Minimum spanning trees (MSTs) of *E. faecium (a)*, *S. aureus* (b) and *K. pneumoniae* (c) isolates based on cgMLST using short read data from two laboratories. Sequencing replicates of identical strains exhibit consistent cgMLST profiles hence leading to direct clustering irrespectively of the executing laboratory. The results for these species were consistent with the findings described for *L. monocytogenes* in the main text. *Data for isolate SA61 are derived from LAB2 and LAB3, because data of this single isolate in LAB5 had too low coverage. The SR sequencing procedure in LAB3 is described below in the Methods section.

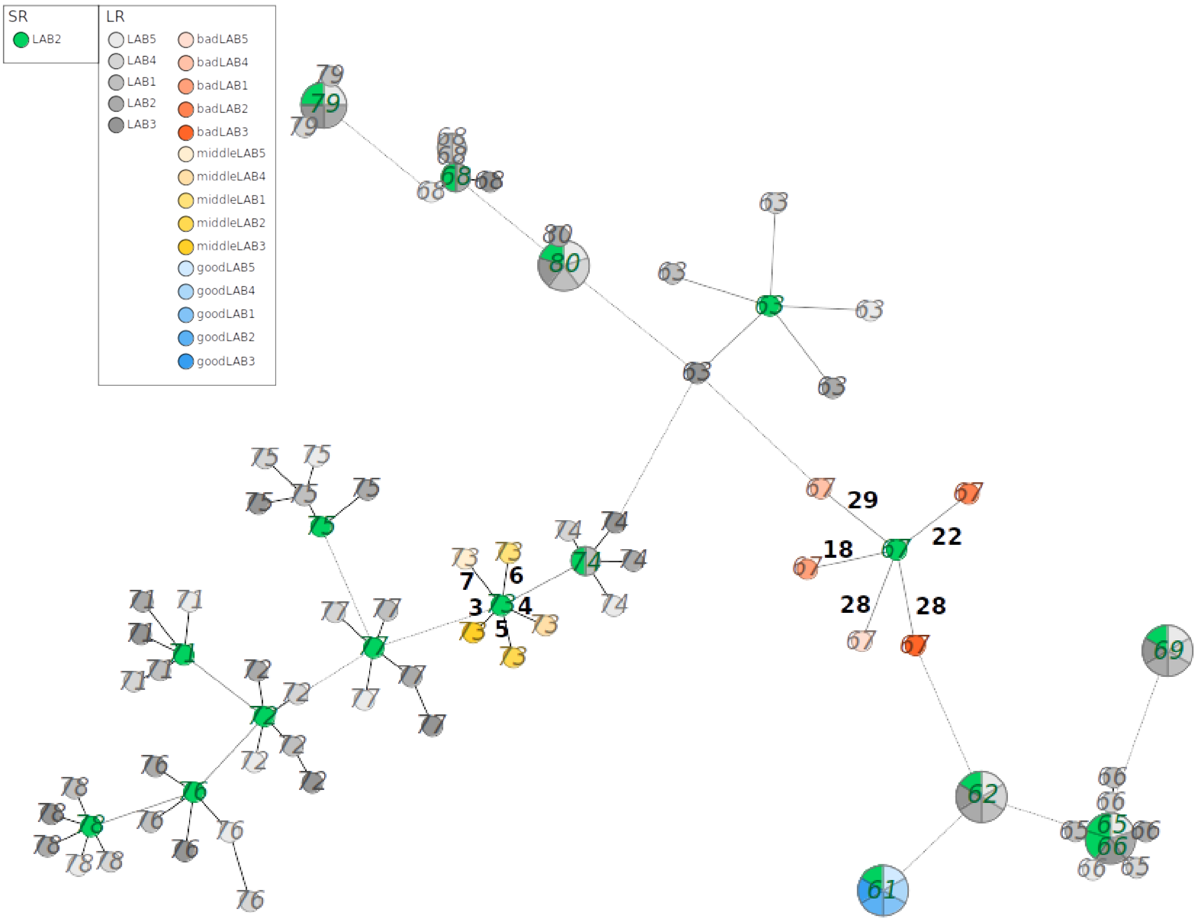

Supplementary Figure 2: cgMLST-based minimum spanning tree (MST) of *S.aureus* using SR (green) and LR data (grayscale). LR assemblies of different participants showed inconsistent typing results depending on the strain under investigation. There are isolates where the typing of the LR matches that of the SR (one exemplary in blue shades), but also others with differences not only to the SR but also between the LR assemblies of the participants. Furthermore, the magnitude of the observed differences varied between isolates e.g. compare one exemplarily shown in blue, yellow and red respectively. For a clearer presentation, we only show the differences for selected strains; the differences at isolate level are detailed in Figure 3 of the main text.

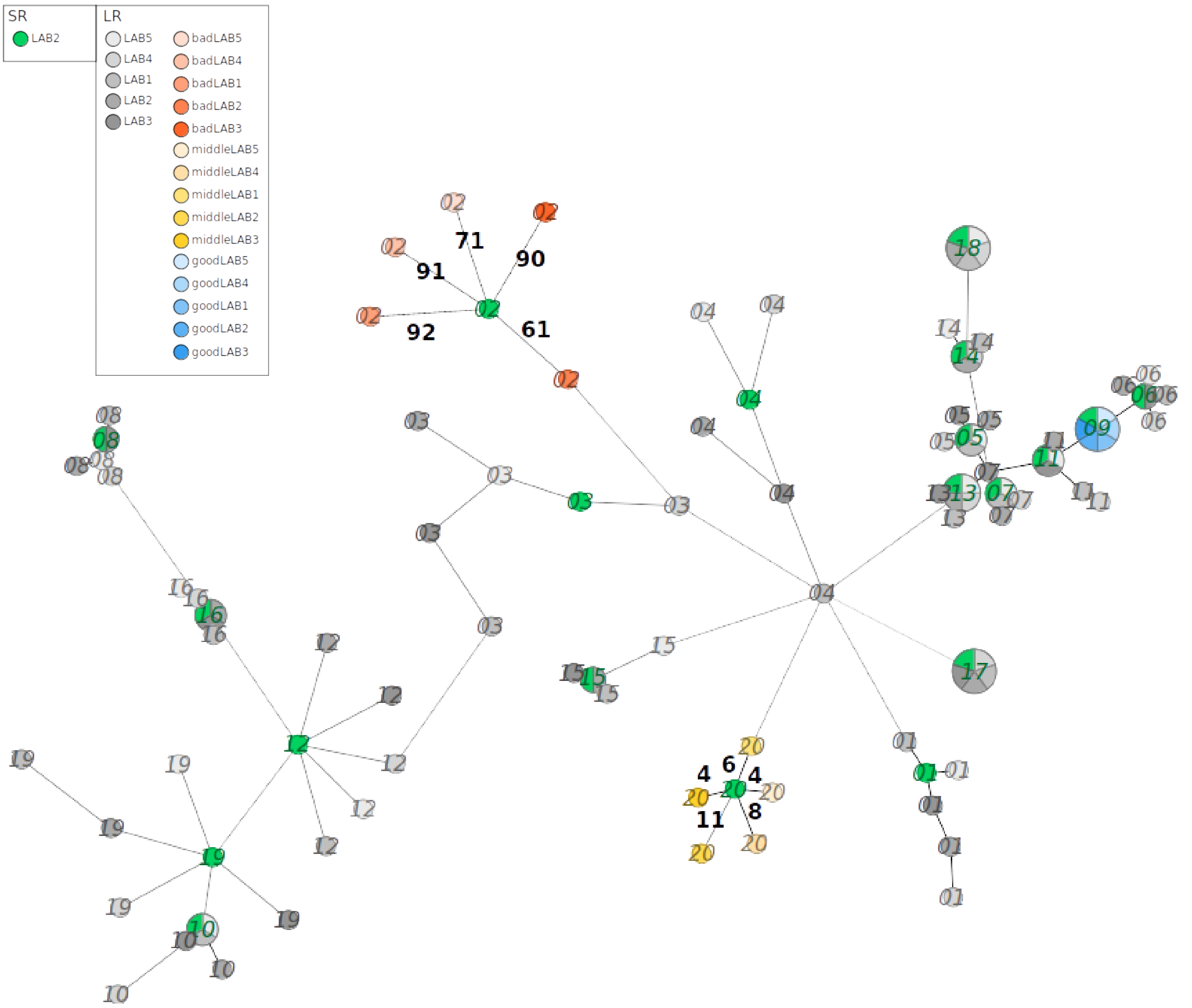

Supplementary Figure 3: cgMLST-based minimum spanning tree (MST) of *K. pneumoniae* using SR (green) and LR data (grayscale). LR assemblies of different participants showed inconsistent typing results depending on the strain under investigation. There are isolates where the typing of the LR matches that of the SR (one exemplary in blue shades), but also others with differences not only to the SR but also between the LR assemblies of the participants. Furthermore, the magnitude of the observed differences varied between isolates e.g. compare one exemplarily shown in blue, yellow and red respectively. For a clearer presentation, we only show the differences for selected strains; the differences at isolate level are detailed in Figure 3 of the main text.

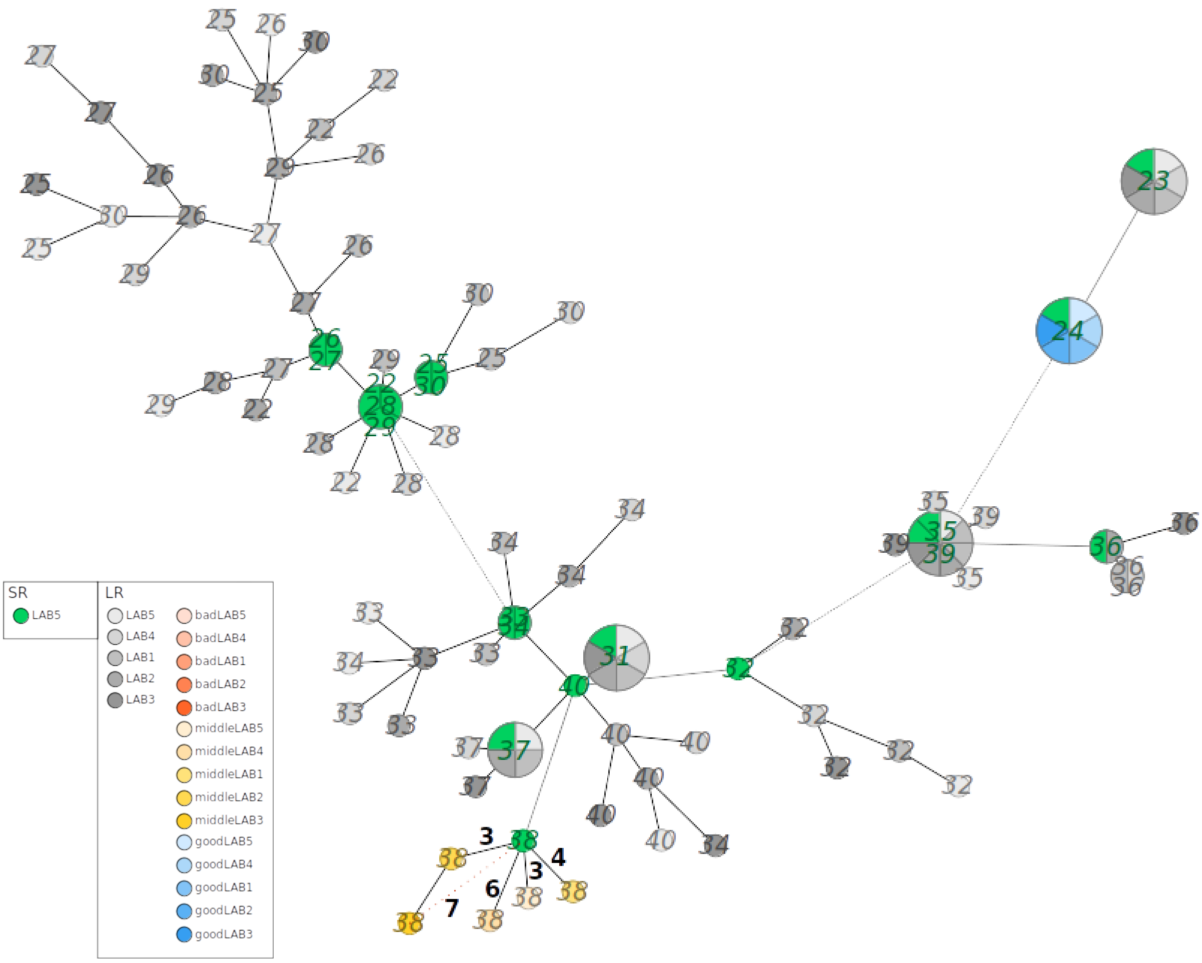

Supplementary Figure 4: cgMLST-based minimum spanning tree (MST) of *E. faecium* using SR (green) and LR data (grayscale). LR assemblies of different participants showed inconsistent typing results depending on the strain under investigation. There are isolates where the typing of the LR matches that of the SR (one exemplary in blue shades), but also others with differences not only to the SR but also between the LR assemblies of the participants. Furthermore, the magnitude of the observed differences varied between isolates e.g. compare one exemplary shown in blue and yellow respectively. For a clearer presentation, we only show the differences for selected strains; the differences at isolate level are detailed in Figure 3 of the main text.

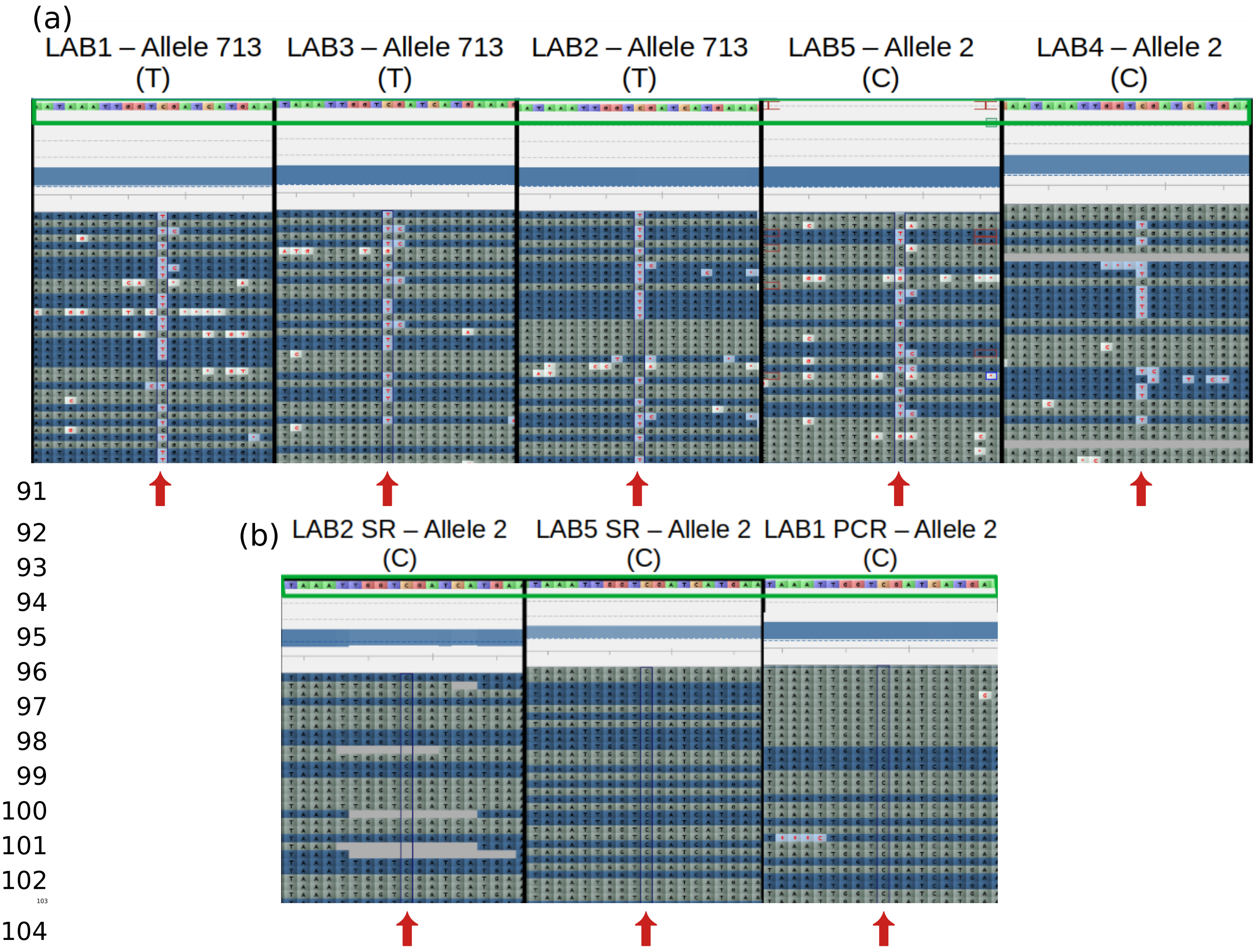

Supplementary Figure 5a: Mapping of the reads of all participants of *E. faecium* isolate 26 at locus 'EF01658', where discrepant typing results between the participants were observed. The difference is due to a single different base in the respective final consensus assemblies, which led to different allele calls in the subsequent allele typing – 'Allele 2', as in the reference or incorrect 'Allele 713'. In all cases, including assemblies with the correct allele variant, the mapping of reads revealed a strand-specific pyrimidine base ambiguity (C or T). This ambiguity reflected the observed difference between the two called alleles and suggested an issue related to strand-specific methylation. Although this error appears at conserved positions at the read level, it accumulated with slight variations and resulted in differences in the final assemblies' consensus sequence and, consequently, in the typing. In agreement with the different typing results, the respective nucleotide frequencies varied among participants: LAB1 (48% C - 50% T), LAB2 (47% C - 53% T), LAB3 (47% C - 52% T), LAB4 (62% C - 38% T), LAB5 (53% C - 44% T). The mapped reads are colored blue or green depending on their strand specificity. The mapping was visualized with Tablet viewer. **5b:** The same mapping as described before but for SR data and whole-genome PCR preamplification LR data of the same isolate showing only the correct reference base. This indicates that the issues in the LR data arise due to DNA modifications.

Different typing results arise even with sub-sampled data under otherwise uniform experimental conditions

The minimal differences in the number of reads with the correct versus the incorrect base led us to hypothesize that this ratio might shift minimally during random subsampling, altering the typing results. To test this hypothesis, we selected one strain with minimal distance to the SR reference and one severely mismatching per species, while ensuring sufficient sequencing depth, to generate two differently subsampled datasets at 100X depth. The assemblies of the subsampled datasets from the previously good performing strains exhibited only negligible deviations from the reference in the typing results. At the same time, assemblies from the problematic strains not only displayed errors relative to the reference but also demonstrated differences in typing between these two assemblies (Supplementary Table 5). This confirms our hypothesis, showing how uncertain/fragile the data situation can be. It indicates an inherent issue in the data, with all preceding steps such as DNA preparation, sequencing, etc., being entirely identical in this comparison. Hence, the mismatches (MM) between assemblies from random subsampling of the data facilitate the identification of problematic assemblies/isolates, as evidenced by discrepancies in typing. Of note: As expected by these findings, typing results also differ when the exact same DNA prep is sequenced again using nanopore (Supplementary Table 6).

Supplementary Table 5: One good and one suspicious strain per species were selected based on the typing mismatches (MM) to the SR reference and high sequencing depth (>120X) for reasonable subsampling. Random subsampling of the original datasets of LAB1 to 100X sequencing depth was based on two different seeds for randomization. Subsequent analysis of the assemblies revealed that the assemblies of problematic strains not only showed a similar number of mismatches with regards to the reference but also between each other, indicating an inherent problem in the data.

	**MM to the Reference**			
	**Subsample 1**	**Subsample 2**	**original result**	**MM between subsamples**
**LM41**	29	28	30	**29**
**LM54**	0	0	0	**0**
**EF22**	4	5	5	**5**
**EF35**	0	0	0	**0**
**KP04**	68	74	73	**53**
**KP13**	0	0	1	**0**

**SA63**	27	29	26	**30**
**SA62**	1	0	0	**1**

Supplementary Table 6: cgMLST mismatches to the SR reference (MM to ref) in assemblies from two sequencing runs on two flow cells in the same lab that relied on the exact same DNA prep of the strains (Rep. – repetition of same DNA). The mismatches between the typing results are in the same range but must not affect the same targets, as shown by the typing differences of the assemblies between each other (Diff. between reps.).

**Strain**	**MM to ref**	**Rep. MM to ref**	**Diff. between reps.**
**KP02**	92	87	**127**
**KP03**	77	76	**82**
**KP04**	73	76	**56**
**KP09**	0	1	**1**
**KP11**	4	4	**0**
**KP12**	55	65	**67**
**KP15**	1	3	**4**
**KP18**	0	0	**0**
**KP19**	65	53	**77**
**KP20**	6	5	**11**

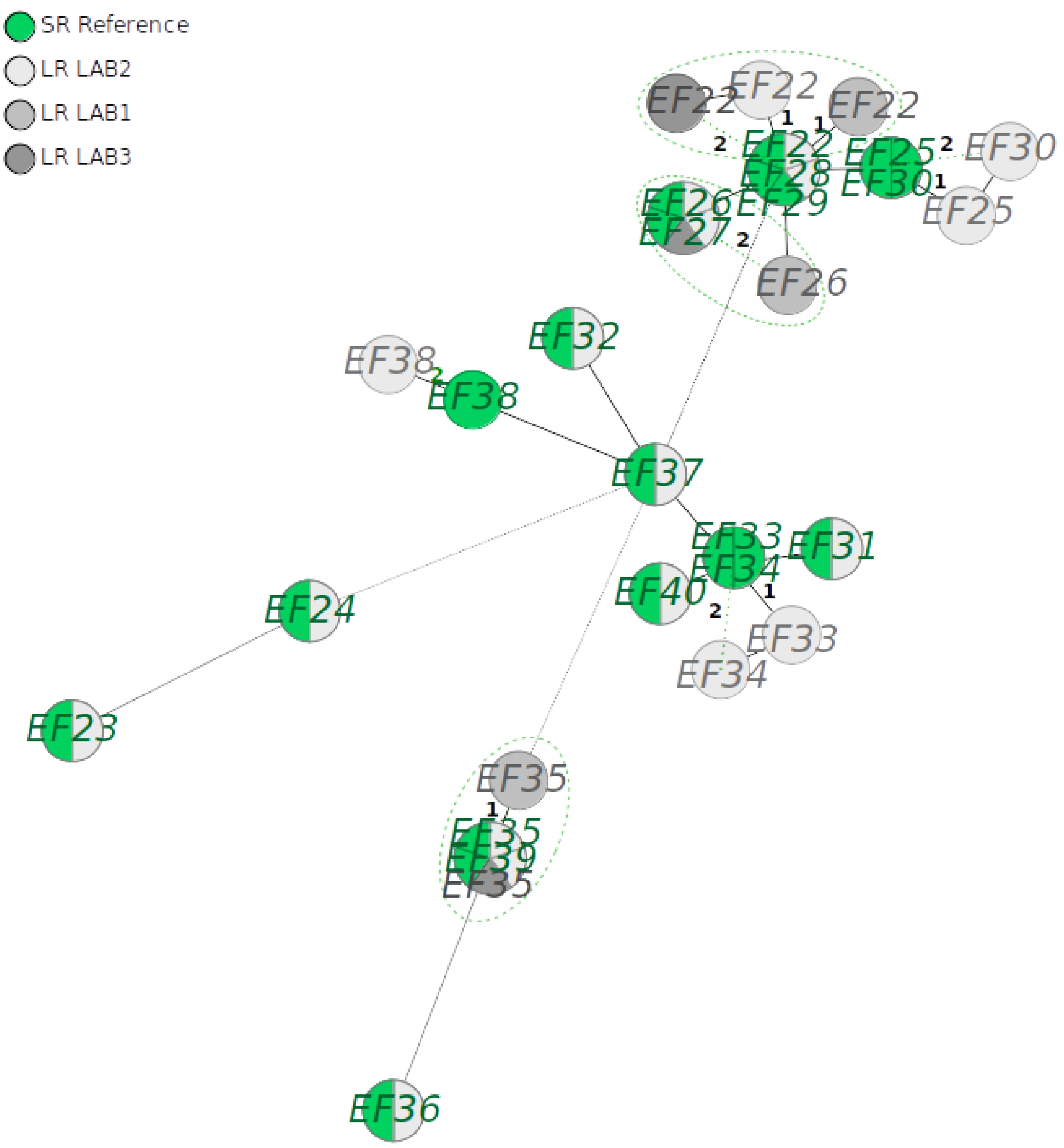

Supplementary Figure 6: The Minimum Spanning Tree (MST) of *E. faecium*, constructed using both short-read (SR - green) reference data and long-read (LR - in grayscale) data under the latest optimal conditions (sampling at 5 kHz, 400 bp/s, bacterial methylation model basecalling, polishing with Racon and Medaka using its variant model), vividly illustrates the significant enhancements achieved in nanopore sequencing. To further assess this progress, LR data from two additional participants were generated and analyzed for 3 selected isolates per species, encompassing one good and two challenging isolates (marked in circles). All distances from the re-sequenced LR assemblies to the reference SR assemblies not being zero are denoted in black numbers.

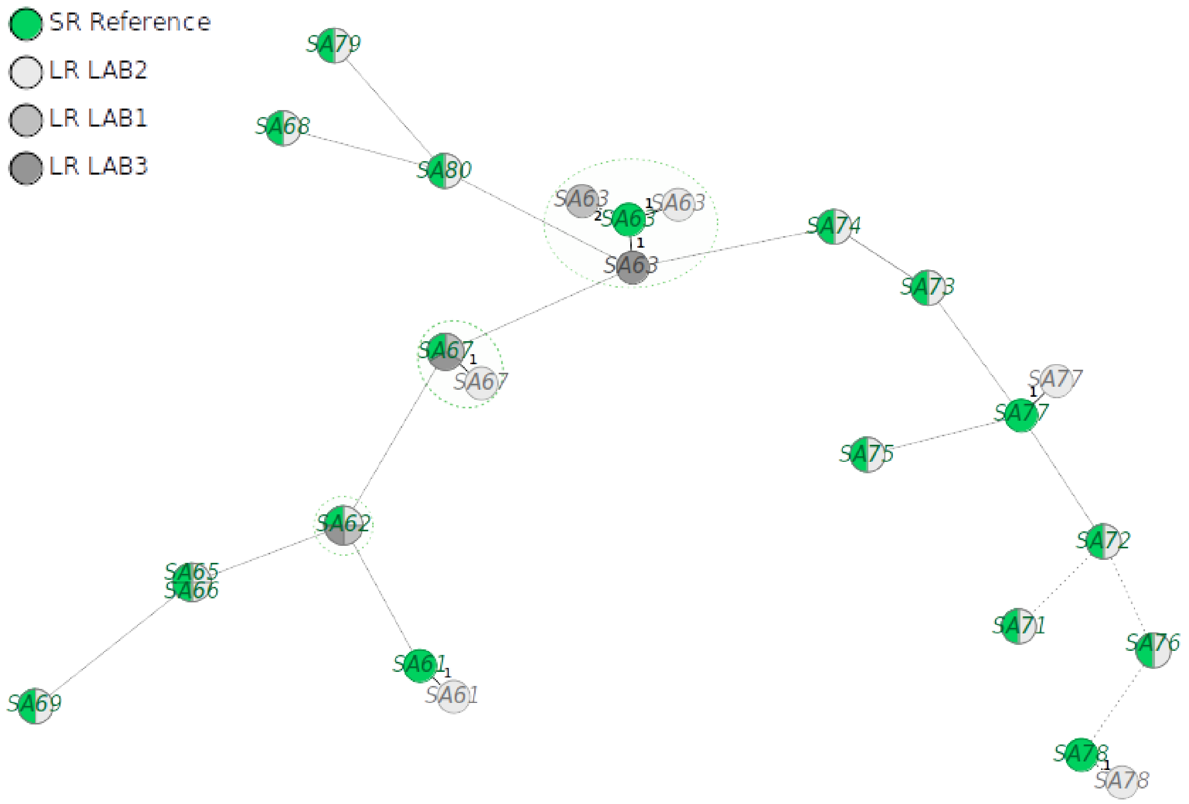

Supplementary Figure 7: The Minimum Spanning Tree (MST) of *S. aureus*, constructed using both short-read (SR - green) reference data and long-read (LR - in grayscale) data under the latest optimal conditions (sampling at 5 kHz, 400 bp/s, bacterial methylation model basecalling, polishing with Racon and Medaka using its variant model), vividly illustrates the significant enhancements achieved in nanopore sequencing. To further assess this progress, LR data from two additional participants were generated and analyzed for 3 selected isolates per species, encompassing one good and two challenging isolates (marked in circles). All distances from the re-sequenced LR assemblies to the reference SR assemblies not being zero are denoted in black numbers.

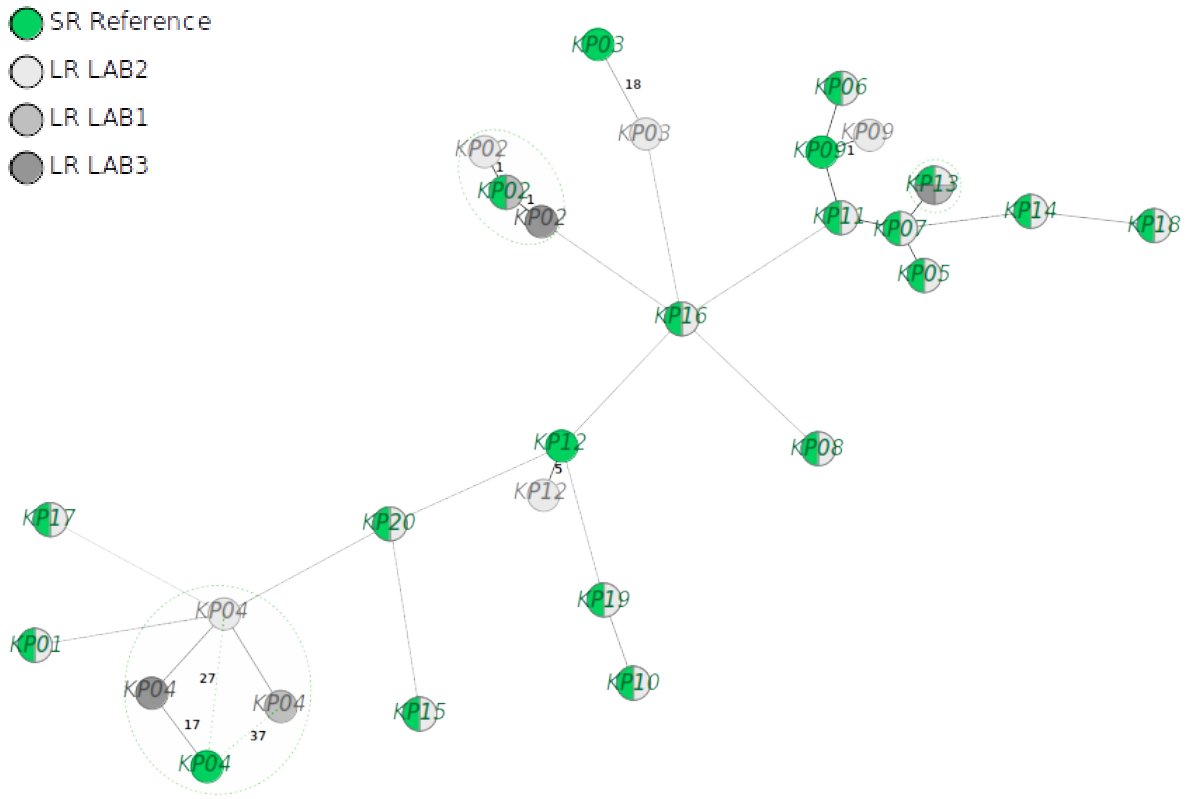

Supplementary Figure 8: The Minimum Spanning Tree (MST) of *K. pneumoniae*, constructed using both short-read (SR - green) reference data and long-read (LR - in grayscale) data under the latest optimal conditions (sampling at 5 kHz, 400 bp/s, bacterial methylation model basecalling, polishing with Racon and Medaka using its variant model), vividly illustrates the significant enhancements achieved in nanopore sequencing. To further assess this progress, LR data from two additional participants were generated and analyzed for 3 selected isolates per species, encompassing one good and two challenging isolates (marked in circles). All distances from the re-sequenced LR assemblies to the reference SR assemblies not being zero are denoted in black numbers.

Methods:

DNA was extracted as described in the methods of the main text for LR sequencing of Lab 3. DNA libraries were generated with the Nextera XT Library Preparation Kit using the Illumina DNA/RNA UD Indexes (Sets A to D) (Illumina, USA). 1ng of input DNA was used. Library concentration was quantified using the Qubit 1X dsDNA Assay Kit (Thermo Fisher Scientific, USA) and the fragment length of the libraries were determined with the Agilent 5300 Fragment Analyzer (Agilent Technology, USA). Sequencing was performed on an Illumina NextSeq 2000 device with the NextSeq 1000/2000 Reagent cartridge of 300 Cycles (Illumina, USA) using 750 pM of prepared DNA library.
